# Association between 21-gene-assay and detection of disseminated tumor cells in patients with early breast cancer: results from the IRMA trial

**DOI:** 10.1007/s10549-023-07031-w

**Published:** 2023-08-09

**Authors:** Léa L. Volmer, Dominik Dannehl, Tobias Engler, Markus Hahn, Christina B. Walter, Markus Wallwiener, Sara Y. Brucker, Florin-Andrei Taran, Andreas D. Hartkopf

**Affiliations:** 1 Department for Women’s Health, University Medical Center Tübingen, 72076 Tübingen, Germany; 2 Department for Gynecology and Obstetrics, University Medical Center Heidelberg, 69120 Heidelberg, Germany; 3 Department for Gynecology and Obstetrics, Freiburg University, 79085 Freiburg, Germany; 4 Department for Gynecology and Obstetrics, Ulm University, 89081 Ulm, Germany

**Keywords:** Disseminated tumor cells, Oncotype DX, Minimal residual disease, Breast cancer, Individualized therapy

## Abstract

**Purpose:**

Disseminated tumor cells (DTCs) in the bone marrow (BM) are known to be of prognostic value for patients with early breast cancer (EBC). In addition to histopathological features, multigene expression assays, such as the commercially available 21-gene Breast Recurrence Score® assay, have been validated for evaluating prognosis and making decisions concerning adjuvant treatment in EBC. In a previous retrospective study from our group, the 21-gene assay was shown to be associated with DTC-detection. A secondary endpoint of the prospective IRMA trial was to evaluate the association between Recurrence Score® (RS) result and tumor cell dissemination in patients with EBC.

**Methods:**

DTC-status and RS result were assessed in patients with ER-positive/HER2-negative EBC with 0–3 pathologic lymph nodes who underwent primary surgical treatment at the Department for Women’s Health of Tuebingen University, Germany.

**Results:**

Patients with a high RS result (≥ 26) were more frequently DTC-positive (22.6%) than patients with a low RS result (8.6%, p = 0.034). The odds for DTC-positivity increased with rising RS values (p = 0.047).

**Conclusion:**

We therefore confirm that a high genomic risk is associated with tumor cell dissemination into the BM. Further trials are needed to investigate whether therapeutic decisions could be further individualized by combining DTC-status and prognostic gene signature testing.

## Introduction

In the era of personalized medicine, decisions concerning adjuvant treatment and evaluations of prognosis are increasingly being based on molecular features of tumors. Particularly in early breast cancer (EBC), multigene expression assays have been validated for survival evaluation [1–3] and treatment decisions [4–6] in estrogen receptor (ER)-positive/HER2-negative tumors. Besides molecular characteristics of EBC, minimal residual disease (MRD) as detectable with the presence of disseminated tumor cells (DTCs) in the bone marrow (BM) is also known to be of prognostic value [7–9]. These DTCs, found in 20–30% of patients with EBC [10], are associated with a poorer outcome, as well as earlier locoregional and distant relapse in breast cancer patients [11, 12].

The commercially available 21-gene Oncotype DX Breast Recurrence Score® assay is already implemented in clinical routine [13–17]. In a retrospective study from our group, the 21-gene assay was shown to be associated with detection of DTCs in patients with ER-positive/HER2-negative EBC [18]. In patients with a Recurrence Score® (RS) result > 18, DTCs were detected more frequently than in those with a RS ≤ 18.

In the **I**mpact of **R**ecurrence Score® (RS) result on adjuvant treatment decisions and tu**m**or cell dissemination in ER-positive and HER2-negative patients with e**a**rly breast cancer (IRMA) trial, RS result and DTC-status were assessed prospectively [19]. This trial already demonstrated that by adding RS results to clinicopathological risk factors in ER-positive/HER2-negative EBC, treatment recommendations could be individualized and over- or undertreatment with adjuvant chemotherapy prevented [19].

A secondary endpoint of this trial was to validate the association between RS result and tumor cell dissemination.

## Methods

### Patients

IRMA is a prospective, monocentric investigator-initiated register study. It was approved by the Ethics Committee of Tuebingen University (789/2018BO2) and conducted according to the guidelines of the Declaration of Helsinki.

Patients who received primary surgery for hormone receptor (HR)-positive/HER2-negative, unilateral EBC (T1–4, N0–1) at Tuebingen University Women’s Hospital, Germany, were included in this study. HR-positive was defined as ER and/or progesterone receptor (PR) expression ≥ 10% according to immunohistochemical evaluation. HER2 status was assessed according to local standards by using the HERCEPT test (DAKO, Denmark). Expression of HER2 was scored on a 0 to + 3 scale. Tumors with a score of + 3 were considered HER2-positive. In case of a score of + 2, HER2 amplification was determined by fluorescence in situ hybridization using the Pathvysion® Kit (Vysis, Downers Grove, IL). Exclusion criteria were recurrent or metastatic disease, extensive lymph node involvement (> 3 positive lymph nodes, based on clinical or pathological status), neoadjuvant systemic therapy, bilateral breast cancer, or a previous history of malignancy.

### DTC detection

For detecting DTCs, BM aspirates (10–20 ml) were collected during surgery for BC and processed within 24 h as described previously [20]. Briefly, mononuclear cells were separated by density centrifugation (Ficoll, 1.077 g/ml, Biochrom, Germany), spun down onto a glass slide (Hettich cytocentrifuge, Germany), and fixed in 4% formalin. Cytospins were stained using the DAKO Autostainer (Dako, Denmark) and a mouse monoclonal antibody directed against Keratin 8/18 Ab-1 (Thermo Fisher Scientific, Fremont, USA) was used. According to the consensus recommendations for standardized tumor cell detection [20], two slides with 1.5 × 10^6^ cells per patient were evaluated after cytokeratin staining. Each batch of samples was analyzed together with leukocytes from healthy volunteers as negative controls and the human breast cancer cell lines MCF 7 and SKBR 3 as positive controls. DTC-positivity was defined as at least one cytokeratin-positive with abnormal cell morphology per 3.0 × 10^6^ cells.

### Assessment of 21-gene Breast Recurrence Score assay

Paraffin-embedded tumor tissue samples were submitted to Exact Sciences (Redwood City, CA, USA), according to guidelines provided by the manufacturer. The 21-gene Oncotype DX Breast Recurrence Score® assay (Exact Sciences, Redwood City, CA, USA) was assessed in all patients. Patients were divided into two groups, based on the RS-repartition of the TAILORx trial: 0–25 (RS low), 26–100 (RS high) [21].

### Statistical analysis

Associations between nominally scaled independent variables were analyzed using the chi^2^-test. Normally distributed data was tested for significance using a two-sided Student’s t-test. The influence of continuous variations in the RS result on DTC status was assessed using logistic regression. Factors that achieved statistical significance with p < 0.05 in the univariate analysis for DTC positivity were assessed by using a multivariate logistic regression. Odds ratios (OR) and confidence intervals (CI) were calculated. All statistical tests were carried out with JMP 16 software 22 (SAS®). Significance level was set at p = 0.05.

## Results

### Patient characteristics

A total of 245 patients were included in the IRMA trial [19]. Among them, BM aspirates were available for 217 patients. Of all patients, 131 were postmenopausal (60.4%). Most tumors were histologically classified as non-special type (75.1%) and were grades 1–2 (83.4%). Around half of tumors were smaller than 20 mm (pT1, 52.1%). Also, the majorityshowed no lymph node involvement (69.6%).

DTCs were detected in 23 patients (10.6%). These findings were associated with tumor size: among patients with a tumor size < 20 mm a 6.2% DTC positivity was observed and among patients with larger tumors this rate was 15.4% (pT2-4, p = 0.027).

DTC-positive patients were more frequently treated with adjuvant chemotherapy (45.5% of DTC-positive patients vs. 24.0% of DTC-negative patients, p = 0.039). ki67 values did not differ between DTC-positive (mean ki67 (%): 20.6, SD ± 8.6) and DTC-negative patients (mean ki67 (%): 18.9, SD ± 11.7, p = 0.398).

The Oncotype DX® test yielded a median RS result of 15 (Q1–Q3: 10–21) with a low RS result (0–25) in 186 patients (85.7%) and a high RS result (26–100) in 31 (14.3%) patients. Recurrence Score result was associated with tumor size and grading: the percentage of patients with a high RS increased with tumor size (p = 0.016) and with grading (p < 0.001) with no patient simultaneously displaying a low grading and high RS result. Patients showing a RS result 26–100 more frequently received adjuvant chemotherapy (93.6% of patients with high RS result vs. 14.8% of patients with a low RS result, p < 0.001) (Table 1).

### Comparison of DTC status and Recurrence Score result

Overall, 31 out of 217 patients (14.3%) showed a high RS result (RS 26–100). As displayed in Table 2, patients with a high RS result (26–100) were more frequently DTC-positive (22.6%) than patients with a low RS result (8.6%, p = 0.034). RS values were higher in DTC-positive (mean RS = 20.2, 95% CI 15.9–24.5) than in DTC-negative patients (mean RS = 16.3, 95% CI 15.0–17.6, t-test p = 0.048).

**Table 1:** Patient characteristics by DTC status and Recurrence Score result

	All patients	DTC-positive		RS ≥ 26	
	n	n (%)	chi^2^ p-Value	n (%)	chi^2^ p-Value
Total	217	23 (10.6)		31 (14.3)	
Menopausal status					
Premenopausal	86	10 (11.6)		12 (14.0)	
Postmenopausal	131	13 (9.9)	0.608	19 (14.5)	0.806
Histology					
Nonspecial type	163	18 (11.0)		27 (16.6)	
Lobular carcinoma	41	4 (9.8)		3 (7.3)	
Other	13	1 (7.8)	0.955	1 (7.7)	0.195
Nuclear grade					
G1	23	2 (8.7)		0 (-)	
G2	158	14 (8.9)		14 (8.9)	
G3	36	7 (19.4)	0.236	17 (47.2)	** < 0.001**
Tumor size					
T1	113	7 (6.2)		10 (8.9)	
T2–3	103	15 (14.6)		20 (19.4)	
T4	1	1 (100)	**0.013**	1 (100)	**0.011**
Nodal status					
N0	151	15 (9.9)		20 (13.3)	
N1	66	8 (12.1)	0.505	11 (16.7)	0.372
Ki67 (mean ± SD)	19.0 ± 11.4	20.6 ± 8.6		29.5 ± 9.1	
Adjuvant therapy					
Chemotherapy	56	10 (17.9)		29 (51.8)	
No chemotherapy	158	12 (7.6)	**0.039**	2 (1.3)	** < 0.001**

**Table 2:** Comparison of Recurrence Score values and DTC status

	All patients	DTC-positive	DTC-negative	
Recurrence Score	n	n (%)	n (%)	p-value (chi^2^-test)
All patients	217	23 (10.6)	194 (89.4)	
Low (< 26)	186	16 (8.6)	170 (91.4)	
High (≥ 26)	31	7 (22.6)	24 (77.4)	**0.034**

In the univariate analysis, the odds for DTC positivity significantly increased by 3.01% for a one-unit increase in RS result (95% CI 0.06–8.32, p = 0.047).

In the multivariate analysis, no significant association was found between clinical or histopathological parameter, RS result, and DTC status (see Table 3).

**Table 3:** Multivariable logistic regression of factors influencing disseminated tumor cell (DTC) detection

Parameter	OR for DTC-detection	95% CI	chi^2^ p-Value
Recurrence Score			
RS < 26	1.0		
RS ≥ 26	2.3	0.8–6.5	0.085
Tumor size			
T1	1.0		
T2–3^a^	2.4	0.9–6.2	0.073
Nodal status			
N0	1.0		
N1	1.1	0.4–2.8	0.890

*OR* odds ratio, *CI* confidence interval, *DTCs* disseminated tumor cells

^a^One case staged as pT4b was excluded from this analysis since this tumor stage is not included in Oncotype DX acceptance criteria

## Discussion

In this study, we compared the results of the 21-gene assay with the DTC status in patients with ER-positive/HER2-negative EBC. To our knowledge, this is the largest prospective trial to date that addresses this question.

We found that DTCs were more frequently detected in patients with a high RS result, indicating that patients with a high RS result may more often be prone to early tumor cell dissemination. These results validate previous retrospective findings from our group [18]. Other studies addressed this question and did not find any correlation between RS result and DTC status [22, 23]. However, in those studies, the overall data were different: Aktas et al. reported that DTCs were detected in 36.5% out of 68 patients who underwent BM aspiration, whereas Singh et al. reported detecting DTCs in 34% of 58 patients included in their study [22, 23]. Both studies reported higher DTC positivity rates than in the largest pooled analysis known to date, in which about 23% of luminal-A-like patients were DTC-positive [11]. In the present study, DTCs were detected in 10.6% of patients, which was a low detection rate in this patient population. The low detection rate in our study is most likely due to the low-risk features of our cohort, with 69.6% patients being node-negative, 60.4% postmenopausal, 83.4% G1-2, and 52.1% with a tumor size < 20 mm. Indeed, 14.3% of the patients had a RS 26–100, which is similar to what was found in the TAILORx trial, [21]. In their study, Singh et al. performed DTC-detection using four more antibodies directed against cytokeratins, which offers a further explanation for the differences in DTC-detection rates [23].

In the multivariate analysis, we did not find a statistically significantassociation between RS result and DTC detection. Although DTC-positivity might be driven by larger tumor size, which was also correlated to RS results, the small sample size may have limited power to detect a statistically significant association between RS result and DTC positivity.

The Oncotype DX Breast Recurrence Score assay evaluates 16 cancer genes, some of which are associated with tumor proliferation and invasion. For instance, Stromelysin 3 (ST3) was shown to be expressed by breast cancer cells that have undergone epithelial-to-mesenchymal transition (EMT) [24] and to promote tumor cell migration [25]. Furthermore, secretion of cathepsins by breast cancer cells has been shown to regulate neutrophils, enabling formation of metastatic niches in the lung [26]. EMT as well as modification of the extracellular matrix and peritumoral milieu have also been reported to be associated with increased tumor cell dissemination in breast cancer [27, 28]. These findings may offer a molecular explanation for the association between high RS values and tumor cell dissemination into the BM.

The IRMA trial previously was able to show that by using RS for decisions concerning adjuvant chemotherapy treatment could be further individualized, mostly reducing recommendations for adjuvant chemotherapy [19]. However, a smaller group of patients was identified who might benefit from chemotherapy according to RS, although it was not initially recommended to them [19]. Until now, the information of DTC-status and RS have not been combined for adjuvant treatment decision.

Major limitations of the current study were the small patient population and the lower rate of DTC-positive patients compared to other studies. These factors may explain the lack of significance in the multivariate analysis and may also limit our ability to evaluate potential associations between RS results and DTC detection in subgroups of interest. Since these data were collected in the years 2018–2020, we are not yet able to present sufficient survival data in such a low-risk cohort.

In conclusion, we confirm that a high genomic risk is associated with tumor cell dissemination into the BM. The Oncotype DX Breast Recurrence Score® assay can identify patients with an increased risk of micrometastatic spread and tumor cell dormancy. Future trials should investigate whether therapeutic decisions could be further individualized by combining DTC detection and prognostic gene signature testing.

## Data Availability

The datasets generated during and/or analyzed during the current study are not publicly available for reasons of patient confidentiality but are available from the corresponding author on reasonable request.
